# Probiotics: Versatile Bioactive Components in Promoting Human Health

**DOI:** 10.3390/medicina56090433

**Published:** 2020-08-27

**Authors:** Javad Sharifi-Rad, Célia F. Rodrigues, Zorica Stojanović-Radić, Marina Dimitrijević, Ana Aleksić, Katarzyna Neffe-Skocińska, Dorota Zielińska, Danuta Kołożyn-Krajewska, Bahare Salehi, Selvaraj Milton Prabu, Francine Schutz, Anca Oana Docea, Natália Martins, Daniela Calina

**Affiliations:** 1 Phytochemistry Research Center, Shahid Beheshti University of Medical Sciences, Tehran 1991953381, Iran; javad.sharifirad@gmail.com; 2 LEPABE—Department of Chemical Engineering, Faculty of Engineering, University of Porto, 4200-465 Porto, Portugal; c.fortunae@gmail.com; 3 Department of Biology and Ecology, Faculty of Science and Mathematics, University of Niš, 18000 Niš, Serbia; zstojanovicradic@yahoo.com (Z.S.-R.); dimitrijevicmarina92@yahoo.com (M.D.); 90anna.aleksic@gmail.com (A.A.); 4 Department of Food Gastronomy and Food Hygiene, Warsaw University of Life Sciences (WULS), 02-776 Warszawa, Poland; katarzyna.neffe.skocinska@gmail.com (K.N.-S.); dorota_zielinska@sggw.pl (D.Z.); danuta_kolozyn_krajewska@sggw.pl (D.K.-K.); 5 Noncommunicable Diseases Research Center, Bam University of Medical Sciences, Bam 44340847, Iran; 6 Student Research Committee, School of Medicine, Bam University of Medical Sciences, Bam 44340847, Iran; 7 Department of Zoology, Annamalai University, Annamalai Nagar 608002, Chidambaram, India; smprabu73@gmail.com; 8 Department of Biomedicine, Faculty of Medicine, University of Porto, 4200-319 Porto, Portugal; francineschutz@yahoo.com.br; 9 Department of Toxicology, University of Medicine and Pharmacy of Craiova, 200349 Craiova, Romania; 10 Institute for Research and Innovation in Health (i3S), University of Porto, 4200-135 Porto, Portugal; 11 Laboratory of Neuropsychophysiology, Faculty of Psychology and Education Sciences, University of Porto, 4200-135 Porto, Portugal; 12 Department of Clinical Pharmacy, University of Medicine and Pharmacy of Craiova, 200349 Craiova, Romania

**Keywords:** nutrition, lifestyle, probiotic properties, evidence based-medicine, mechanisms, clinical studies, safety

## Abstract

The positive impact of probiotic strains on human health has become more evident than ever before. Often delivered through food, dietary products, supplements, and drugs, different legislations for safety and efficacy issues have been prepared. Furthermore, regulatory agencies have addressed various approaches toward these products, whether they authorize claims mentioning a disease’s diagnosis, prevention, or treatment. Due to the diversity of bacteria and yeast strains, strict approaches have been designed to assess for side effects and post-market surveillance. One of the most essential delivery systems of probiotics is within food, due to the great beneficial health effects of this system compared to pharmaceutical products and also due to the increasing importance of food and nutrition. Modern lifestyle or various diseases lead to an imbalance of the intestinal flora. Nonetheless, as the amount of probiotic use needs accurate calculations, different factors should also be taken into consideration. One of the novelties of this review is the presentation of the beneficial effects of the administration of probiotics as a potential adjuvant therapy in COVID-19. Thus, this paper provides an integrative overview of different aspects of probiotics, from human health care applications to safety, quality, and control.

## 1. Introduction

A frequent occurrence of lifestyle diseases and negative lifestyle habits in today’s society has unquestionably triggered the development of food science and nutrition.

The current trend for healthy and rational eating is related to the increase in nutritional awareness among consumers, influencing the functional food sector at the same time. The sector includes natural and processed foods containing desirable biologically active molecules that, when administered in correct quantitative and qualitative proportions, provide clinically proven health benefits [1,2,3,4,5,6].

This kind of food has an unquestionable impact on the prevention, management, and treatment of chronic diseases in modern society provided that it is of a balanced nutritional value, proper composition, and endangers neither the health nor the life of a consumer [7].

Metabolomics studies have increasingly focused on the research directions in food and nutrition, specifically addressing food composition, physiology of healthy microbiota, and the nutrition and health relationship [8,9,10,11].

Probiotics can be defined as dietary supplements that contain live microbial strains capable of persisting in (or transiently colonizing) the human intestinal tract and confer a beneficial influence on host physiology, such as improving health. This process is particularly important when the normal, native flora has been disturbed: at this time, exogenously supplemented probiotics of a species/strain can temporarily colonize the intestinal tract and stabilize the microflora composition, thus restoring vital physiological function—a commensal flora [12].

The mechanisms of action of probiotics are diverse: they produce antimicrobial substances such as organic acids or bacteriocins, regulate the immune response through the secretion of IgA against possible pathogens, reduce the risk of developing an allergy, improve the function of the intestinal mucosal barrier, increase the stability or promote the recovery of the commensal microflora when it is disturbed, modulate the expression of host genes, release functional proteins such as lactase or natural enzymes, and decrease the adhesion of pathogens [13].

In addition to these probiotic properties, in the context of the current COVID-19 pandemic, new studies have shown that probiotics can be used in the prophylaxis and adjuvant therapy of this disease caused by SARS-CoV-2 [14,15].

Therefore, the use of probiotics to improve health is based on the following principle: exogenous microorganisms (from food sources) amplify the beneficial physiological effects of normal (indigenous) intestinal microflora.

According to the current state of knowledge, probiotic microorganisms show a number of beneficial properties to the human body, on multiple levels [16,17], and all are made safe by representing a Generally Recognized As Safe (GRAS) status, are acid- and bile-tolerant and able to adhere to the intestinal tract as well as colonise it. Moreover, probiotics are capable of protecting the host from noxious microorganisms and of strengthening the immune system [18]. However, both in the field of science and industry, the term “probiotics” is mainly narrowed to the group of lactic acid bacteria (LAB) [19].

Although microorganisms used in food production have a long history of safe use and are frequently familiar as “food grade” or GRAS microorganisms, the Joint Food and Agriculture Organization of the United Nations (UN) and World Health Organization (WHO) Expert Consultation on Evaluation of Health and Nutrition Properties of Probiotics started to establish several guidelines [20], for both monitoring and evaluating food probiotics. The first meeting was held in Canada (2002), and the team recognized and described the essential information to correctly validate health claims, besides establishing a set of mandatory assays to assess probiotics strains’ safety [20].

**Figure 1:**
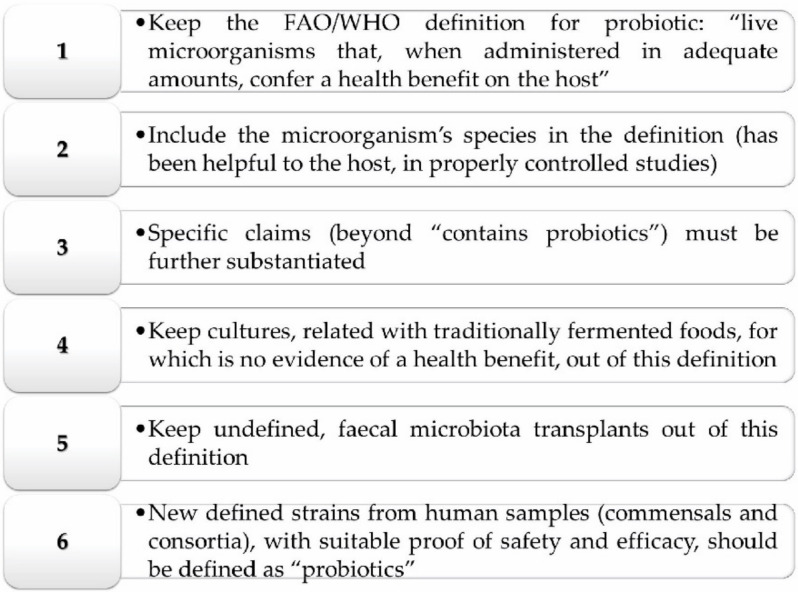
The new concept of “probiotic”. Abbreviations: Food and Agriculture Organization of the United Nations (FAO), World Health Organization (WHO).

Nonetheless, these regulations are not in law in any country, as noteworthy disparities are found among worldwide jurisdictions such as the European Community, Canada, and the United States. Since this meeting, a lot of clinical scientific data have triggered the development of probiotic-rich products in the market, and many misappropriations of the term “probiotic” have been reported. For that reason, in 2013, the International Scientific Association for Probiotics and Prebiotics (ISAPP) created the new concept of “probiotic” [21], as briefly pictured in Figure 1.

Unlike probiotics, which designate living microorganisms, bacteria mostly colonize the gastrointestinal (GI) tract; prebiotics are fibers that cannot be absorbed or broken down by the body and therefore serve as a food source for probiotics, especially the genus *Bifidobacterium*, increasing their number. They are indigestible food ingredients that beneficially affect the host by selectively stimulating the growth and/or activity of a limited number of bacteria in the colon, which can improve the health of the host [22].

Prebiotics are found naturally in foods such as artichokes, garlic, and onions, among others. It may be necessary to consume a large amount of these foods to have a “bifidogenic” effect—increasing the level of friendly bacteria in the intestine. For this reason, many people find it easier to take a prebiotic supplement or a combination of probiotics and prebiotic supplements (called symbiotics) to ensure that they reach the level of friendly bacteria [23].

Research shows that there are different types of prebiotics, in the same way that there are different types of probiotics. In prebiotics, the key differentiating factor is the length of the chemical chain, the short chain. The middle chain or the long-chain determines where in the GI tract the prebiotic has an effect and how the host can feel the benefits [22].

Common prebiotics includes inulin, fructooligosaccharides (FOS), galactooligosaccharides (GOS), lactulose, and lafinose. Prebiotics from FOS are low-molecular-weight carbohydrates and only promote the growth of probiotics [24]. Prebiotics from GOS are short-chain oligosaccharides, resistant to the digestive process in the upper GI tract, with the following roles: i) support the selective development of beneficial cells in the intestinal microbiota; ii) maintain the structure and functions of the digestive system; iii) maintain immune balance by the development of lymphoid tissue associated with the intestine (GALT) that stores immune cells; iv) support the balance of the intestinal flora, which can be influenced by various factors (e.g., diet, lifestyle, stress) [25]. The advantage of prebiotics is that they act synergistically with probiotics with symbiotic action and it is the most suitable option when an optimal repopulation of the flora is desired [24,25].

In this sense, and given the above-highlighted aspects, the present review article aims to provide a critical overview of probiotics use, drifting from its human health care applications to safety and quality control, viability, and safe consumption for clinical purposes.

## 2. Methodology

Scientific search engines PubMed, Scopus and ScienceDirect were searched to retrieve literature and cross-references using keywords: “probiotics”, “prebiotics”, “mechanism of action”, “gastrointestinal disorders”, “depression”, “carcinogenesis”, “immunostimulatory effect”, “adjuvant therapy COVID-19”, “food matrices”, “probiotics viability”, “side effects”, “quality control”. Written papers in English, meta-analyses, and research articles and clinical trials were included. Short communications, abstracts, letters to the editors, studies that contained, and the administration of homoeopathic medicines were excluded.

## 3. Effectiveness in Promoting Health: Mechanism of Action of Probiotics

**Figure 2:**
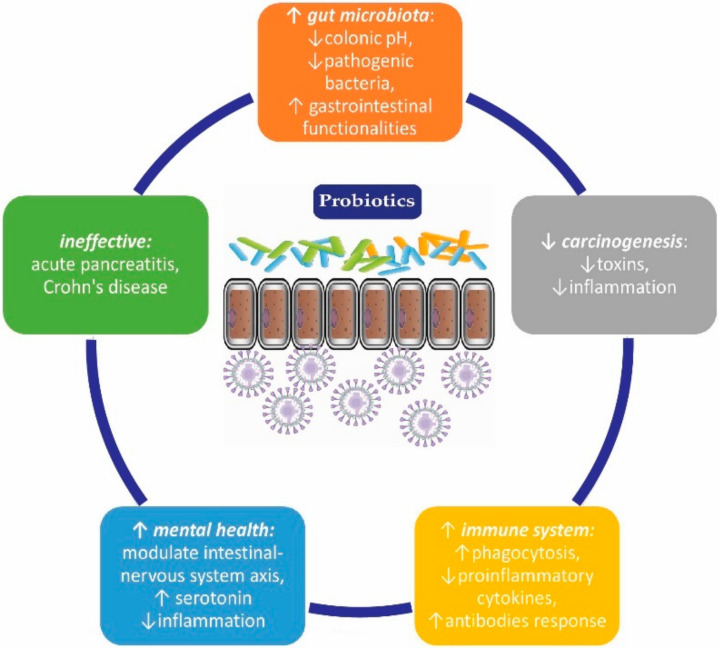
The most important beneficial properties of probiotics in promoting human health.

Probiotic preparations are also frequently used by healthy individuals, for preventive purposes, to obtain beneficial health effects and improve quality of life (Figure 2).

It has been observed that the microbiota has a variety of impacts on health and disease. The intestinal microflora is composed of microbes that are usually found in the intestine but can be affected by diet, lifestyle, exposure to toxins, and antibiotic therapy [2]. There is a relationship between health, disease, the immune system, and changes in the microbiota. It is believed that harmful bacteria begin to predominate in various disorders, such as obesity, autoimmune diseases, and infections. Probiotics play a role in maintaining the immune balance in the GI tract through direct interaction with immune cells. Thus, probiotics have become a tool to counteract “dysbiosis” by replacing the harmful microflora with beneficial microorganisms [26].

Most of the mechanisms behind the beneficial effects of probiotics are not yet known, but they are known to be multifactorial and to differ by species. Some of these mechanisms are related to the effects of probiotics to antagonize various harmful microorganisms through secretion of antimicrobial substances, competition for adhesion to the mucosa and epithelium, a strengthening of the intestinal epithelial barrier, and modulating the immune system.

### 3.1. Use of Probiotics in Gastro-Intestinal (GI) System Disorders

The probiotic’s effects on a human organism are extensive, and the clinical examination proves unquestionable beneficial effects of selected microorganisms in the treatment of GI system disorders.

#### 3.1.1. Acute Infectious Diarrhea

At present, few therapeutic options change the course of the evolution of an acute infectious diarrheal disease, the treatment being generally a symptomatic one. Current guidelines encourage the association of probiotics in the treatment regimen. These have shown favorable results only in the case of enterocolitis of bacterial etiology; the results being inconsistent in viral infections. The administration of probiotics should be started at the onset of diarrheal disease and, although there is no evidence to support the duration of administration, it is recommended to continue for 1–2 weeks from the resolution of symptoms [27].

A meta-analysis of 63 clinical trials, including 8014 children and adults with acute infectious diarrhea, found a significant reduction in the duration of the disease with the use of probiotics (25 hours less), a 59% decrease in the risk of prolongation over four days and a decrease in the number of daily stools. Another meta-analysis, which included 17 studies and 2012 children with acute diarrhea, showed a decrease of approximately 20 h in the duration of the disease in patients receiving probiotics [28].

The effectiveness of probiotics has also been proven in the case of traveler’s diarrhea. Thus, in a meta-analysis of 12 clinical trials and 5171 patients, a 15% reduction in the risk of developing traveler’s diarrhea was observed in the case of prevention by probiotic administration starting two days before the trip and continuing during the trip [29].

However, a clinical study, which enrolled 646 children with acute diarrhea generally caused by rotavirus infection, did not show a significant difference between the group of patients who received the probiotic containing *Lactobacillus rhamnosus* GG and the control group in terms of concerns the frequency of stools, the duration of diarrheal disease, the presence of vomiting, or the duration of hospitalization [30].

#### 3.1.2. Diarrhea Associated with Antibiotic Therapy and Caused by *Clostridium difficile*

One of the most significant health benefits brought by mentioned microorganisms is their bactericidal and bacteriostatic action towards pathogens. It is the lactic acid that performs the bactericidal function. It is able to neutralize the electrochemical potential of cell membranes, cause intercellular protein denaturation, and lower the pH level of pathogenetic cell cytoplasms. Other chemical compounds produced by probiotics that are able to inhibit pathogenetic bacteria growth and development are acetic acid, acetaldehyde, hydroperoxide, and specific peptides similar to antibiotics, called bacteriocins. The bacteria that are particularly sensitive to the activity of hydroperoxide are anaerobic of the *Clostridium* genus. Bacteriocins can inhibit Gram-positive pathogenetic bacteria growth, including *Listeria* sp., *Staphylococcus* sp., and *Streptococcus* sp. [31,32].

Regulating the proportions of gut microflora composition, probiotic microorganisms prevents or lessens the course of diarrhea. This property is of particular significance, especially in cases of diarrhea resulting from travelling effects and acute, chronic infectious diarrhea caused by viruses and bacteria of *Clostridium difficile*, *Shigella,* and *Salmonella* sp., and some *Escherichia coli* strains. The presence of probiotics in a digestive tract contributes to the reduction of a carrier state of pathogenic bacteria mentioned above as well as to the prevention of their adhesion to a gut lining. Supplying an organism with probiotic bacteria has a special function in the regeneration or formation of a gut microflora, which is crucial after antibiotic treatment or radiotherapy [33,34]. Experiments on mice infected by parasites show a similar preventative mechanism of probiotic effects, i.e., immune response stimulation and tight adherence to a gut lining. It was proven that probiotics are capable of preventing infections caused by parasitic *Toxoplasma gondii* and *Cryptosporidium* [35,36].

Probiotics are effective in the prevention and treatment of diarrhea associated with antibiotic therapy and in the prevention of diarrhea caused by *C. difficile* infection [37]. The administration of probiotics should be started on the first day of antibiotic therapy and continued for 1–2 weeks after the end of antibiotic therapy [38,39].

A meta-analysis of 23 studies looked at the effect of probiotics containing *L. rhamnosus* or *Saccharomyces boulardii* in preventing antibiotic-associated diarrhea in 3938 children. There was an 11% decrease in the relative risk of developing diarrhea in those who received probiotics from the control group, including diarrhea associated with *C. difficile* infection, as well as reduced stool frequency, accelerated recovery, and reduced disease duration [40].

#### 3.1.3. *Helicobacter pylori* Infection

The results are inconsistent in terms of the effectiveness of probiotics as an adjunct to antibiotic therapy to eradicate *Helicobacter pylori* infection. A meta-analysis of 8 clinical trials, which included 1163 patients, concluded an increase in the rate of eradication of *H. pylori* infection in combination with probiotics containing *Lactobacillus*. However, a meta-analysis of 21 clinical trials in 3542 patients did not reveal an improvement in the rate of eradication of the infection in combination with probiotics compared to placebo [41].

#### 3.1.4. Inflammatory Bowel Diseases (IBD)

It is believed that it is possible to achieve beneficial effects by manipulating the microbiota in patients with inflammatory bowel disease [10,42]. Thus, the effectiveness of treatments aimed at dysbiosis present in inflammatory bowel diseases is being investigated. However, from the currently available data, it cannot be concluded whether the use of probiotics has a general benefit in the evolution of these diseases.

Probiotics are effective in increasing the remission rate in adults with ulcerative colitis, but not in maintaining remission. The administration should be started at the onset of exacerbation of colitis and continued for 1–2 weeks after the resolution of symptoms. A meta-analysis of 23 clinical trials involving 1763 patients, showed a 23.3% increase in the remission rate in patients with active ulcerative colitis when using probiotics [43].

Probiotics are a promising treatment option in irritable bowel syndrome, but the quality and quantity of existing evidence is quite low [44]. A meta-analysis of 23 clinical trials, which included 2575 patients with irritable bowel syndrome, showed a significant improvement in overall symptoms, bloating, and flatulence when using probiotics. Another meta-analysis of 21 studies (1639 patients) also showed an improvement in symptoms and quality of life compared to the control group [45].

#### 3.1.5. Chronic Constipation

Probiotics are effective in children and adults with constipation. The administration should be started at the onset of symptoms and continued for as long as symptoms persist. In a meta-analysis of 2 clinical trials (165 patients with chronic idiopathic constipation), a significant increase in the average number of stools per week was observed in patients treated with probiotics, compared to the control group [46].

#### 3.1.6. Necrotizing Enterocolitis

Probiotics reduce the risk of necrotizing enterocolitis and death in infants. Therapy should be initiated in children at risk of developing this disease and continued for as long as the risk remains high. A meta-analysis showed that the administration of probiotics significantly reduced the risk of severe necrotizing enterocolitis (effect observed in a group of 5529 patients) and mortality (effect observed in a group of 5112 children) [47].

#### 3.1.7. Hepatic Encephalopathy. Nonalcoholic Steatohepatitis

Probiotics have shown favorable effects in hepatic encephalopathy [48]. A meta-analysis of 6 clinical trials, which included 496 adults with cirrhosis, showed a 15.3% reduction in the relative risk of developing hepatic encephalopathy when given probiotics. In another meta-analysis of 21 studies involving 1420 patients, improvement in recovery and quality of life was observed with the use of probiotics, but without effect on mortality.

A systematic review of 3 clinical trials concluded that the administration of probiotics improves liver function in adults with non-alcoholic steatohepatitis based on biological markers, but lacks data on the effects on patients’ clinical condition [49].

#### 3.1.8. Celiac Diseases, Non-celiac Gluten Sensitivity, Food Allergies

Wheat has been a staple in the human diet for over 10,000 years [50]. The response to lifestyle and dietary protein in wheat can cause a variety of symptoms and immune responses. Gluten is a family of proteins found in cereals, often involved in several digestive pathologies, especially in early childhood. The spectrum of gluten-induced pathologies is increasing as new pathogenic mechanisms are understood and described. Three heterogeneous gluten-induced situations were identified: celiac disease (an autoimmune mechanism), wheat allergy (an allergic mechanism), and non-celiac gluten sensitivity (non-autoimmune, nonallergic). If the first two (celiac disease and a wheat allergy) have already become classics, being described, studied, and understood, the third entity—non-celiac gluten sensitivity—is a topic of attention as a new disease or syndrome with gluten intolerance [51].

Celiac disease is an autoimmune condition that manifests itself in inflammation of the small intestine caused by gluten consumption. Celiac disease (celiac disease) is the only autoimmune disease in which both the trigger, the epigenetic factor (gluten), but also the specific HLA antigen (human leukocyte antigen) are known. Unfortunately, celiac disease is underdiagnosed, and people with or without GI manifestations, symptomatic and asymptomatic subjects can be found [52]. Dysbiosis can promote celiac disease, and recent studies have established a link between dietary gluten (wheat, oats, barley, rye) and changes in the intestinal microbiome. Several studies suggest that modern lifestyle and eating habits may play a role in disrupting the balance of intestinal bacterial flora [53].

Thus, it has been shown that the intestinal microbiome is different in terms of composition in celiacs compared to healthy people. For example, the decrease in the number of *Firmicutes* bacteria and the increase in Proteobacteria have been found in both children and adults with celiac disease [54].

Other studies have reported a decrease in the population of protective, anti-inflammatory bacteria such as *Bifidobacterium* and an increase in *Bacteroides* and *E. coli* bacteria in patients with active celiac disease. Moreover, in these patients, low diversity and an altered metabolic function were discovered, which is associated with a decrease in the concentration of protective acids—short-chain fatty acids [55]. Studies have shown that mice colonized with healthy flora are protected from gluten-induced pathology, while mice colonized with *Proteobacteria* develop moderate gluten-induced pathology, and those colonized with *E. coli* from patients with celiac disease develop this disease [56].

Non-celiac gluten sensitivity is a new entity, introduced at the time of describing the gluten-induced pathology in individuals with symptoms similar to those of celiac disease, but without the presence of specific antibodies and intestinal lesions found in celiac disease. The mechanism of the disease is not elucidated. A possible role of wheat-induced innate immunity is suggested, as well as altered intestinal permeability, with excessive absorption of gluten-derived peptides. No predisposing genetic factor was identified. Recent data suggest the major role of the innate immune system through an abnormally born wheat-induced response. There is an increased expression of innate immunity markers (toll-like receptor TLR-2), increased CD3^+^ intraepithelial T cell density, and altered intestinal permeability (“leaky gut”), which lead to excessive absorption of gluten-derived peptides [57].

Food allergies can sometimes be fatal if the immune system overreacts. The foods involved in the most significant allergic reactions are peanuts, hazelnuts, walnuts, fish, seafood, milk, eggs, wheat, soy, and seeds [58]. The common mechanism that leads to various food allergies is a lack of immunological and clinical tolerance to ingested food, leading to immediate or acute reactions, mediated by specific antibodies, immunoglobulins E (IgE), or delayed clinical symptoms by a mechanism mediated by specific immune cells [59]. Sensitization (production of IgE antibodies) to food allergens can occur through the GI tract, skin, and, less frequently, through the respiratory tract. Currently, the “double exposure theory” suggests that allergenic food sensitization occurs through low-dose skin sensitization, especially when the skin barrier is affected, while the early introduction of food proteins induces oral tolerance [59].

Wheat allergy is an immune reaction to any of the hundreds of types of protein that are in wheat and that occur within minutes of the ingestion of food. A person allergic to wheat needs to avoid any product that contains wheat but tolerates other foods that contain gluten (rye, barley) [51].

The researchers found that the bacteria *C. difficile*, part of the intestinal flora, usually protects against peanut allergy, which is very common among the population and is currently incurable [60]. In a recent study, researchers genetically modified mice to make them allergic to peanuts. So, the first group of mice was raised in a sterile, germ-free environment, the second group of mice was treated with antibiotics shortly after birth, thus massively reducing their intestinal bacterial flora and another group of mice were raised normally. Mice in the first two groups had very strong immune reactions to peanuts, producing high levels of antibodies compared to mice whose intestinal flora was normal. This exaggerated sensitivity to peanut allergens could be greatly attenuated by reintroducing a mixture of *Clostridium* bacteria into the intestine of rodents [61].

On the other hand, the introduction of another type of intestinal bacteria, of the genus bacteroids, did not lead to any results, which shows that *Clostridium* bacteria play a unique role in protecting against allergies. Genetic and molecular analysis of *Clostridium* bacteria has shown how these microorganisms prevent allergic reactions, triggering a mechanism inside immune cells that produces large amounts of a molecule known to reduce intestinal wall permeability [62].

Peanut-allergic mice in the two study groups were treated either using the molecule, IL-22, or by introducing *Clostridium* bacteria into the gut [63]. In both cases, a clear decrease in the amount of allergens in the blood was observed. Researchers have shown that *Clostridium* bacteria are common to the human intestinal flora and are an obvious target for therapies for preventing or treating food allergies [63]. These results highlight the effectiveness of probiotic therapies, live microorganisms, bacteria or yeast, which, added to certain foods, such as yogurt or cereals, would have health benefits, by inducing an immune response that prevents allergens from entering the bloodstream, these bacteria minimize the body’s exposure and prevent sensitization. This key mechanism leads to the development of allergies.

#### 3.1.9. Symptomatic Uncomplicated Diverticular Disease

Uncomplicated symptomatic diverticular disease is a clinical condition characterized by chronic digestive symptoms such as recurrent abdominal pain, abdominal distension, and altered intestinal transit, secondary to the presence of diverticula. The clinical picture is similar to that present in irritable bowel syndrome, the semiological features that differentiate the uncomplicated symptomatic colonic diverticular disease being represented by pain located predominantly in the left iliac fossa, pain that persists over 24 h; stools are frequently diarrhea, and symptoms do not resolve after defecation or flatulence [64].

The main objective in the management of the uncomplicated symptomatic diverticular disease is the control of abdominal symptoms. No therapeutic standard has been defined; however, optimal pharmacological and nutritional strategies for the management of uncomplicated symptomatic diverticular disease have been described.

Targeted therapeutic strategies have been developed on the intestinal microbiome: administration of dietary fiber, probiotics, or rifaximin. The therapeutic effect of fiber is not fully known. However, its administration by diet or in the form of supplements is encouraged to alleviate the symptoms of uncomplicated diverticular disease [65].

Their properties can explain the beneficial effect of fibers: they increase the mass of residue, stimulating intestinal transit, and act as prebiotics in the colon by promoting the proliferation of sanogenic species of intestinal flora: *Bifidobacterium* and *Lactobacilli*. The intestinal flora changes rapidly depending on changes in diet. However, there is no clear evidence of the therapeutic benefit of a high-fiber diet in the therapeutic control of the diverticular disease [66].

Following the idea of modulating the activity of the intestinal microbiota, the use of probiotics has recently been tried for the treatment of uncomplicated symptomatic BD, but especially for the prevention of disease recurrences [65]. For the time being, however, the results are not very conclusive, the data being quite discordant. The most commonly used strains belonged to the genera *Bifidobacterium* and *Lactobacillus*, but strains of *E. coli* or other microorganisms, such as *S. boulardii*, were also used. What is certain is that certain probiotics for certain groups of patients with uncomplicated symptomatic bowel disease (BD) may bring some benefits. Still, in the absence of well-structured studies, it is difficult to say whether or not these benefits are comparable to those obtained from the use of therapeutic solutions (rifaximin-a, soluble fiber). As a result of the evidence, they have accumulated over time [66].

In Table 1 is summarized the data regarding the efficacy/ineffective effects of probiotics in GI diseases.

### 3.2. Mild and Moderate Depression

Preclinical and clinical studies have suggested relief of depressive symptoms when using probiotics [68]. Favorable effects are thought to be mediated through the intestinal-nervous system axis and consist of reducing inflammation and increasing serotonin levels [69,70].

According to a recent study, probiotics may be effective in reducing depressive symptoms in previously untreated patients with mild to moderate forms of depression. Patients were treated with probiotics containing two strains known to act on the intestinal-nervous system (*Lactobacillus helveticus* and *Bifidobacterium longum*). They showed a significant reduction in mood and sleep disorders after the use of probiotics for 4 weeks. The results were maintained at 8 weeks after treatment. The results, although preliminary, are important because probiotics have the advantage of not having the many side effects of antidepressant drugs [71].

### 3.3. Potential Effect in Reducing Carcinogenesis

Probiotic bacteria are capable of degrading and bonding carcinogenic compounds found in a diet or produced by pathogens. Among the compounds responsible for carcinogenesis we can classify enzymes such as nitroreductase, glucuronidase, and some mycotoxins. The experiments on animals demonstrate that probiotic bacteria can inhibit cancer cells growth, such as of abdominal hernia, sarcoma and leukemia. It is acknowledged that consuming a probiotic aliment may result in a decrease in the occurrence of colon cancer cases [72].

Probiotics play a significant role in neutralizing cancer, nonetheless, an accurate mechanism of anticancer action of probiotics has not been fully understood yet. In fact, this field of science requires in vivo models and clinical trials to assess the anti-cancer potential of probiotics. Presently, it is assumed that microorganisms having probiotic properties hinder the processes of the formation of noxious compounds of protein transition or fat oxidation through the inhibition of pathogen microorganisms’ growth [73]. What requires particular attention is the products of protein transition, including carcinogenic, mutagenic or teratogenic biogenic amines and nitrosamines found in fermented meat products, such as raw aged meat.

Nutritional controversy of used nitrate III in meat technology is a subject of controversy as it is capable of forming toxic nitrosamines in situ and in vivo. Nitrosamines have carcinogenic properties to many species of animals. Moreover, they display mutagenic, teratogenic, and embryotoxic action [74].

N-nitrous compounds may arise in food products during technological processes, food storage, and directly inside a human body as a result of the activity of digestive tract microorganisms, which are responsible for reducing nitrate V to nitrate III, oxidizing ammonia to nitrites, and degrading proteins to the II-row amines. These N-nitrous compounds, which can occur in food during technological processes are an important risk factor for gastric cancers [75]. The majority of nitroso-compounds are formed in the process of endogenous synthesis. The bacterial reduction is started in the mouth and is continued in the stomach through its low pH level. Nitroso-compounds formed in a stomach, are able to diffuse over long distances through permeation to other organs. The large intestine is also an environment of unfavorable chemical reactions undergone by amino acids, amides, indoles, phenols, and glycocholic acid. Probiotic starter cultures added to food should inhibit or reduce negative reaction from the perspective of proteolytic transition, which influences the security of finished products [76].

### 3.4. Immunostimulatory Effect

Regarding the role that the immune system plays in maintaining and improving health, from a traditional point of view, immunity is seen as a defense system against intrinsic factors (neoplasms and tumors) and extrinsic factors, causative agents of diseases (pathogens). On the other hand, this definition presents only a part of the whole.

By controlling and orchestrating the immune response, the immune system is also able to regulate inflammatory events and control and limit the progression of certain pathologies. This occurs mainly through the production of modulating hormones (cytokines) that are able to shape and modify the character of an immune or inflammatory reaction. In this context, it should be obvious that the microbes present in the intestine are not just passive residents of the mucosa of the GI tract. Most likely, the signals generated by the interactions between the microorganisms of the GI tract and the immune system are responsible for most of the beneficial effects of probiotics on health [77].

Probiotic bacteria are capable of stimulating specific and nonspecific defense mechanisms in a human body. The use of probiotics is recommended when the immunity of an organism weakens, which lowers the risk of the occurrence of inflammation, allergies, or infection [78,79].

Probiotic preparations have proven their beneficial influence in the clinical course of eczema among children allergic to the proteins present in cow milk. The research has also indicated that traditional treatment with the use of an elimination diet resulted in health improvement among over a half of tested children, while using probiotics increased the percentage of children exhibiting health improvement to 90% [36,80]. Breast-fed babies provide another example of the stimulating effect of probiotics on the immune system. *Bifidobacterium* sp. detected in the feces of children resulted from the increased level of IgA antibodies, which did not relate to the babies fed with formula milk [81]. A suitable probiotic supplementation is also recommended to pregnant women and babies under 12 months old [82].

Song et al. [83] demonstrated the anti-allergic potential of *L. plantarum* L67 and its application to yogurt. Immunomodulation within a human body is one of the most completely elucidated aspects of the health-enhancing effects of probiotics.

Additional studies on *L. rhamnosus* GG have shown that probiotics can attenuate immune-mediated atopic manifestations by supplementing infant or maternal nutrition, may partially control the immune-mediated inflammatory response in adults (by regulating the expression of leukocyte inflammation receptors), and may reduce the incidence and severity of diarrhea in infants while increasing the number of circulating antibodies [84].

Probiotics are able to modulate the immune system via immunostimulation and immunoregulation, and thus have a significant impact on health and disease status, which have an intrinsic immune component. In the case of immunostimulation, probiotics can provide an amplification of the immune response in key aspects of effector mechanisms specific for combating infectious diseases or intrinsic pathologies, such as the development of neoplasms. In addition, the ability of probiotics to stimulate cytokine secretion can provide a very important regulatory function in controlling conditions characterized by immune dysfunction, such as chronic inflammation and allergies [85].

Administration of fermented dairy products containing *Lactobacillus johnsonii* La1 or *Bifidobacterium lactis* Bb12, for 3 weeks resulted in an improvement in the phagocytosis capacity of leukocytes in the peripheral blood. The increase in phagocytosis was sustained for several weeks after discontinuation of probiotics, with granulocytes exhibiting higher phagocytic activity than monocytes. Increases in the expression of various receptors involved in phagocytosis have also been observed in neutrophils, phagocytic index, or bactericidal capacity [86].

Aging is associated with immunodeficiency, suggesting that probiotics may correct the decline in phagocytic cell function associated with aging. *L. rhamnosus* and *B. lactis* resulted in a significant increase in phagocytic activity of neutrophils and monocytes, especially in subjects with impaired immune function prior to surgery.

It was observed that phagocytic activity is correlated with age, subjects over 70 years of age having a greater improvement in phagocytic activity than those under 70 years of age following probiotic supplementation. Several studies have reported an improvement in the activity of NK (natural killer) immune cells and the percentage of NK cells in the peripheral blood in subjects who received probiotics, as in the case of phagocytic activity.

Numerous studies have demonstrated the ability of certain strains of probiotics to potentiate the humoral immune response to infections. In children with rotavirus-induced diarrhea, administration of *Lactobacillus* GG resulted in a significant increase in IgG, IgA, and IgM levels [86].

Stimulation of post-vaccination immunogenicity was also observed. Administration of *B. bifidum* and *L. acidophillus* La1 resulted in a 4-fold increase in the IgA autoimmune response following immunization for *Salmonella typhi*, while *Lactobacillus* GG improved the response to the oral rotavirus vaccine. Certain strains of probiotics have improved the immune response to immunization with the polio virus: *L. rhamnosus* and *L. paracasei* have stimulated the autoimmune response, especially IgA and the secretion of specific anti-polio IgG and IgA immunoglobulins. These observations suggest the potential adjuvant effects of probiotics in improving vaccine efficacy [87].

The protective effect of the Shirota strain of *L. casei* in the recurrence of colon cancer after surgery has been demonstrated. The increase in the percentage of helper T and NK lymphocytes in adults with colorectal cancer suggests that the effect of *L. casei* on immune function plays an important role in suppressing tumor cell development. The host immune response can be stimulated by *B. longum* subsp *infantis* 35624 *(B. infantis),* causing tumor suppression or regression [88].

The mechanisms by which probiotics influence the immune response are not fully understood and known. It is estimated that certain probiotic molecules are recognized by specific receptors in immunocompetent cells (monocytes, macrophages, dendritic cells, etc.), causing the release of inflammatory cytokines. Numerous studies have also reported increased IL-1, IL-2, IL-6, IL-10, IL-12, IL-18, TNF-α, and γ-interferon production due to in vitro leukocyte stimulation as a result of prebiotic supplementation. Other evoked effects of probiotics on the immune system are regulation of the ratio of T-helper1 / T-helper 2 cells; and a stimulation of the proliferation of intraepithelial lymphocytes [89].

### 3.5. The Potential of Probiotics in Prophylaxis and Adjuvant Therapy in COVID-19

In January 2020, the World Health Organization (WHO) stated that there is an emergency situation related to the outbreak of an infection caused by a new type of virus in the coronavirus family [90]. The new type of coronavirus was named SARS-CoV-2, and the disease was downgraded by infection with this virus to COVID-19. With the exception of Remdesivir, there is currently no approved treatment or vaccine for COVID-19 [91]. In COVID-19 disease management, a therapeutic strategy involves antiviral, anti-shock, anti-hypoxemia, anti-infective, maintenance of the electrolyte and microbiota balance. Multidisciplinary and individualized treatment was applied to each to increase the therapeutic effect [92]. Thus, researchers around the world have conducted various studies in search of possible adjuvant treatments such as natural bioactive compounds, vitamins, and trace elements.

A recent study showed that SARS-CoV-2 behaves like a bacteriophage, meaning a virus that infects bacteria [93]. Genetic sequencing of microflora from six severely infected COVID-19 patients in the same family showed the presence of significant amounts of *Prevotella* in the feces [94]. This could explain the large variations in viral load from one test to another in the same person. The hypothesis is that the virus infects these bacteria, which then cause inflammation, sometimes fatal. Infections involving *Prevotella* are already known to cause acute respiratory symptoms. If COVID-19 is proven to be a mixed infection, both viral and bacterial, then the benefit of combining hydroxychloroquine and azithromycin is scientifically supported. In particular, azithromycin attacks *Prevotella* and intracellular microbes [95]. Several patients experienced a marked decrease in bifidobacteria and lactobacilli in the intestine. They were given probiotics and prebiotics to restore balance and prevent risk of secondary infections [96]. Antibiotic therapy has been reserved for people with a long history of disease and repeated fever. The *L. plantarum* strain, acting on the intestinal mucus, made it possible to prevent epithelial cell infection by coronaviruses in an animal model study [97].

In chronic inflammatory diseases, ingestion of a high concentration probiotic complex also allows a reduction in the plasma level of proinflammatory cytokines and an increase in the level of cytokines that regulate inflammation, with changes in the fecal microbiota compared to the control group [77]. Another probiotic complex, using Lactobacillus brevis as the dominant strain, showed similar results, but acting on the intestinal–brain axis by immune, metabolic, and nervous pathways [98]. It is noteworthy that Lactobacillus brevis, a species of lactic acid bacteria, is able to prevent *Prevotella* from forming biofilms. These biofilms are one of the means used by bacteria to protect themselves from the immune system and antibiotics [99]. Finally, obese people, who are more affected by Covid-19, have a microflora that is even richer in *Prevotella* [100].

If the administration of probiotics to this population gives results in terms of improving the markers of obesity (fat mass, blood sugar, insulin, etc.), these results are even more visible to individuals in whom the bacteria were present.

### 3.6. Diseases in Which Probiotics are Ineffective

Probiotics are ineffective in acute pancreatitis and Crohn’s disease. A meta-analysis of 6 clinical trials evaluating 536 patients with severe acute pancreatitis showed that the use of probiotics did not lead to significant changes in the rate of infection, the total number of infections, the rate of operations, the duration hospitalization, or mortality. Insufficient evidence was also recorded in the case of 3 analyses on the efficacy of probiotics in patients with Crohn’s disease in order to induce remission, maintain remission, or prevent the postoperative recurrence rate [67].

## 4. Probiotics as Pharmaceutical Dietary Supplements

Among the probiotic microorganisms that exhibit a prevailing occurrence in food, pharmaceutical industries encountered Lactic acid bacteria (LAB) belonging to the *Lactobacillus* and *Bifidobacterium* genera. Probiotic LAB used in food fermentation are safe for consumption with food as well as in the form of dietary supplements. However, it is noteworthy that the effectiveness and health benefits depend not exclusively on the strain of probiotic microorganisms, but also on the geographical origin of a strain and on the place of a living population taking the probiotic [101].

The related literature reveals the marked influence of isolation environment on suitable microorganisms’ selection for probiotic preparations: dietary supplements, as well as starter cultures for food production. In the conducted scientific research, it was proven that the gut microbiota can be characterized by a unique constitution depending on the origins of an organism, both human and animal, taking into account such aspects as various geographical areas, climate zones, or eating habits [102,103].

Furthermore, literature data indicate that multi-strain dietary supplements are at an advantage over single microorganisms, because combining several strains may expand the range of preparation properties, such as by broadening the spectrum of antimicrobial action [104,105]. What is worth noticing is the fact that probiotics provided in a form of dietary supplements or within food are not medicinal products. Their influence on an organism and health benefits depend on several factors, including the source of a strain, with spontaneously fermented food being particularly beneficial to the human digestive tract, as well as geographical origins of a strain, supplemented population match, and production process, like food additives, humidity, pH, fermentation, and storage or freeze-drying conditions [36,106].

Simone et al. [107] suggested in a recent study that the probiotic products contain not only live bacteria but also dead cells, their fragments and even molecules. During the process of fermented food or pharmaceutical supplements production, dead bacteria and their fragments cannot be separated and removed from live probiotic cells. Thus, further studies are needed to show whether the presence of dead bacterial cells is positive, negative, or neutral to the health and nutritional value of such products [105,108].

Scientific studies conducted worldwide reveal that probiotic bacteria supplied with food adapt easier to the GI tract environment and have better health potentialities than those consumed through a form of pharmaceutical preparations [7,109,110]. In the EU countries, consumers can easily access multiple probiotic preparations. However, only few of them show scientifically confirmed beneficial health effects. Moreover, the majority of such products’ labels contain neither the proper nomenclature of probiotic species nor a strain for the recommended dose conferring therapeutic effects [36,107].

Fermentation in the small intestine lowers the pH level, which increases the absorption of calcium and other mineral constituents. The results of scientific research suggest that probiotic bacteria exhibit therapeutic effects on hepatic encephalopathy as well as antagonizing *H. pylori* growth, the major cause of gastric and duodenal ulcers. Probiotic supplementation may favorably affect the treatment of peptic ulcer disease, particularly due to the use of antibiotics [16,17]. In summary, in addition to the listed probiotic properties, in the recent literature can be found information about anti-diabetic, anti-obesity, anti-inflammatory and angiogenic activity of probiotics microorganisms [16,111,112]. Additionally, an important phenomenon is the effect of probiotics on the human brain, namely on the synthesis of neurotransmitters that helps to suppress anxiety and depression [16,113].

In recent years, knowledge on the human microbiome, especially connecting human resident microbes and physiology, has become very important. The field of the nutritional value of food is influenced in part by a person’s gut microbial community (microbiota) and its component genes (microbiome). Probiotic foodstuffs have been shown to cause alteration in the human gut microbial content and to have a positive impact human health [80,114,115]. Undoubtedly, what is really important for the proper functioning of an organism is the intestinal microbiota composition as well as its replenishment mainly through the consumption of food products containing selected bacteria strains.

## 5. Particular Food Matrices and their Protection Role on Probiotic Viability

It is acknowledged that probiotic strain administration needs to be prudently calculated. First, it is essential to analyze if fermented or not-fermented food could be more appropriate to stimulate the required effect. Then, to determine whatever food matrix, i.e., raw material to which probiotics can be placed to be accepted as a food product, will be more suitable. Actually, the description of: (i) specific probiotic strains, (ii) food matrix, and (iii) dietary content interaction with probiotics are the latest research topics for food technology and industrial experts [116].

Hence, to evaluate the food matrix, it is crucial to assess the technological skills needed to get a sensorial accepted product and to take into consideration the protective function of probiotics through their passage in the digestive tract. In both cases, the most critical is to preserve alive the highest number of probiotic bacteria, which will rely on a matrix’s chemical composition and its physical state. The food substrate composition (e.g., carbohydrates, fat, proteins) and pH can disturb the growth, stability, and survival [7] of probiotic microorganisms during GI transit. Numerous matrices are vehicles for delivering probiotic bacteria in GI and are considered the basis for probiotic food development [117].

Introduced probiotic bacteria can origin fermentations or be in the not-fermented product. Traditionally, the probiotics used in dairy beverages is broadly extended and milk is the most popular food matrix. Despite milk containing the required growth factors for probiotic growth, it does not necessarily mean that they are available in the assimilable forms, nor in optimal doses. On the other hand, several studies have reported that vegetable and fruit juices or pulp addition (prebiotic) may have deleterious effects on probiotic strain viability, due to its acidity and mostly by the presence of antimicrobial compounds (i.e., phenolic compounds) [110]. For instance, many factors may affect *Lactobacillus* sp. and *Bifidobacterium* sp. viability in dairy beverages, such as the probiotic strains used, pH, the presence of hydrogen peroxide and dissolved oxygen, the metabolite concentration (lactic and acetic acids), the medium buffering capacity, storage temperature, and the nature of added ingredients [7,110]. The main strategies to overcome this problem involves the dairy beverage’s supplementation with whey protein concentrate, that has a higher buffering capacity compared to caseins subsequently delaying the post-acidification during storage. In addition, sulfur amino acid release in heat treatment of whey may lessen the redox potential, producing a positive effect on probiotic survival [110].

Other dairy foods are considered good vehicles for *Lactobacillus* and *Bifidobacterium* species, such as hard or semi-hard cheese. The only problem can be salt concentration, so the viability and activity must be guaranteed at the time of consumption. In several studies, *L. acidophilus, L. plantarum*, and *L. pentosus* have been largely introduced into pasteurized hard and semi-hard cheeses as the sole starter. These reports have shown that probiotic survival during the storage period declined when storage time and/or salt concentration are augmented [118].

In some cases, fermented milk products are fermented by monocultures of probiotic bacteria, but routinely supporting cultures are applied to speed up the acidification process and to deliver the desired texture and flavor. Several *Lactobacillus* sp. and *Bifidobacterium* sp. survive in fermented milk products for 4–8 weeks under refrigeration. Casein glycomacropeptide, amino sugars, and oligosaccharides support these probiotics’ growth, leading to the development of probiotic foods [119].

In the research of Neffe and Kołożyn-Krajewska [120] and Neffe-Skocińska et al. [121], it was found that *L. casei* ŁOCK 0900 and ŁOCK 0908 strains, added to cured pork loins, reached a total of 10^7^ log CFU/g, while in samples with 0.2% glucose, the total count was 10^8^ log CFU/g. In addition, and considering the storage conditions, the authors found higher LAB bacteria growth, including *L. casei* ŁOCK 0900, in pork loins during the 21-day ripening period at 20 °C (from 10^7^ to 10^8^ CFU/g) and the lowest in loin samples aged at 16 °C (from 10^4^ to 10^6^ CFU/g). Moreover, Wójciak et al. [122] revealed that *L. casei* ŁOCK 0900 and *L. paracasei* ŁOCK 0919 may be effectively used as ingredients in fermented sausage production.

Meat products-containing probiotic production is still in development and is much more problematic than producing other probiotic products, as they have the characteristics of a raw material. In fact, meat products create a suitable environment for microbiota growth, so lately multiple efforts have been made to use probiotics in these products. Conversely, meat probiotic products manufacture demands surpassing some technological limits, such as the meat native microflora, the need to employ certain additives (e.g., nitrites, salt), lower the water activity, and lower the amount of natural sugars.

The dry meat matrix consists of meat and fat which “encapsulate” bacteria, protecting them from the critical passage of the GI tract. Moreover, they are typically not or are only slightly heated, being thus suitable for probiotic carriage into the human GI tract. All this promotes a buffer effect in bacteria [123,124].

The quality traits of these kind of products is narrowly connected to the maturing during drying; the long-term storage and growth of probiotic microorganisms evidently has a great impact on its sensorial, nutritional, safety, and other features. In fact, the key matter is that these strains should be present at similar levels to have the desired health potential on consumers’ gut flora. Klingberg et al. [125] showed how important the LAB use was, also indicating the high preservation of CFU of viable cells, both at the time of consumption and during the storage time. Some authors signposted that probiotic bacteria could be better adjusted at the meat environment than at LAB ones [124].

Likewise, changes in pH also pose a survival challenge for fermented meat products containing probiotics. A reduction in pH from 5.6 to 4.9 post-fermentation limits probiotic survival (*L. rhamnosus* GG and E-97800) in fermented sausage over fermentation and ripening processes [126]. Thus, fermented sausages can be manufactured using *L. acidophilus* and *B. animalis* strains, although in the case of *Bifidobacterium* spp. their use as a starter culture has led to several complications such as its low content post-fermentation and its absence after long storage periods (60 days). The opposite scenario is found with the *L. acidophilus* strain when used as a starter culture: high counts (10^6^ CFU/g) and viable for more than 60-day storage [127]. On the other side, probiotic bacteria sensitivity to curing agents (i.e., sodium chloride and sodium nitrite) is also challenging in meat fermentation, although several strategies have been applied to overcome this issue. For example, the use of UV irradiation triggered the generation of *L. gasseri* mutants able to resist these compounds [128], but microencapsulation has also shown to be promising [129].

Considering that probiotics are applied as additives in food, so as to deliver several health benefits, several studies have also been conducted to determine the in vitro adhesion of several LAB probiotic strains to intestinal cells, it being found that *L. plantarum* strains have larger adhesion rates to intestine intimae than those isolated from a sausage (*L. brevis* and *L. paracasei*) [130]. In cereals and legume fermentation, probiotics have also been increasingly used, given its richness in sugars, that can be used as carbon sources by probiotic strains. Specifically, the ability of cereal-based products to maintain probiotic growth is related to their concentration in fibers (e.g., xylooligosaccharides, xylan, arabinoxylan), a growth substrate for probiotics. Moreover, cereals also have a high content in minerals, vitamins, sterols, and other growth factors, supporting the probiotics’ growth, including LAB [117]. More recently, soy products have been assessed as potential probiotic vehicles. In fact, soy has been evidenced to be an outstanding raw material on non-dairy probiotic food development [116]. In addition, peanuts have also been recently studied for probiotic food formulation [131].

An interesting novel approach would be to incorporate probiotics in a dry food matrix. Yet this involves more distinct challenges than liquid probiotic products. A 2004 report described the detection of *B. lactis* in the feces of healthy human volunteers after consumption of an oat-based cereal bar containing *B. lactis* Bb-12 [132]. As main conclusions, the authors stated that dry food matrices keep the probiotic quality equal as dairy products and may then extend probiotic use and shelf-life.

Still considering fruits and vegetables as key nutrient sources (i.e., minerals, vitamins, dietary fibers, and antioxidants), they have been stated as perfect substrates for probiotics growth, with good acceptability rates between all age groups [110]. In this sense, fermented vegetable and fruit juices containing probiotics have been increasingly designed for therapeutic purposes. Since consumers’ demands for non-dairy-based probiotic products have largely been augmented, vegetable or fruit juice-based probiotic drinks have reached a relevant status. Actually, many native LABs have been identified in raw vegetables, such as *L. plantarum, L. paracasei, L. casei, L. delbrueckii*, and *L. brevis*, isolated from natural vegetable lactic acid fermentation. However, the use of probiotic cultures in these foods are, indeed, a great challenge given the above-listed constrains. At large, probiotics growth and viability in fruits and vegetable drinks hang on elements such as bacterial species and strains used, pH, and lactic and acetic acid concentration in the final product [110].

However, as has already been explained, it has been reported that these foods are, in fact, good matrices for probiotic growth. Peeling and cutting vegetables raises the minerals, sugars, vitamins and other nutrients released from the cellular content, which creates a good environment for microbial growth [133]. For instance, carrot juice seems to be a good growth medium for both *L. rhamnosus* and *L. bulgaricus growth*, with a significant load post-fermentation (10^9^ CFU/mL) [110].

Carrot juice is reported to naturally have ≈2% (w/v) of sucrose, 1% (w/v) of glucose and 0.8% (w/v) of fructose, the main carbon and energy sources for probiotic growth. Additionally, carotenoid content (α-and β-carotene) is partially affected by probiotic bacteria metabolism over fermentation [110,134,135]. Moreover, cabbage has been shown to be a good medium for probiotics’ growth [136], it being even proposed by Jaiswal and Abu-Ghannam that probiotic cabbage juice consumption can be a functional beverage for vegetarians and/or dairy food-allergic consumers [137]. Indeed, fresh cabbage juice revealed a high viable cell count of *L. brevis, L. plantarum*, and *L. rhamnosus* (9–10 log CFU/mL), during weeks of storage. In another report from the same authors, *L. plantarum* C3, *L. casei* A4, and *L. delbrueckii* D7 demonstrated to also be able to rapidly grow in sterilized cabbage juice without any nutrient supplementation [137].

Nonetheless, given the higher acidic pH in these products and consequently the high vulnerability of some probiotic strains, microencapsulation appeared as a key strategy to offer protection to acid-sensitive probiotics, since encapsulated probiotics survived over 6 weeks of cold storage with more than 10^5^ CFU/mL or g, as free probiotic cells lost their viability within 5 weeks. Besides, complementing these foods with prebiotics is also a good strategy to improve probiotics’ viability and stability [117]. Thus, microencapsulation in sealed capsules of different materials have been the most widely used, releasing their content under specific environments. Among these materials, lipids such as oil emulsions, milk fat, and water insoluble microcapsules appear to be of high interest. Furthermore, a combination of two different approaches have appeared as a feasible solution [138].

Additionally, the use of a food matrix naturally containing a high content of ingredients with protective effects can also be implemented. As the lipid fraction of cocoa butter has shown to protect bifidobacteria, the feasibility of using chocolate as a carrier for a microencapsulated mixture of probiotic *L. helveticus* CNCM 1-1722 and *B. longum* CNCM 1-3470 has also been assessed, and the results indicated the association of a chocolate coating with microencapsulated probiotic strains as an excellent solution [138]. In fact, it is known from the medical point of view that low-moisture foods can act as efficient carriers for small amounts of pathogens, thus leading to human infections, as was also shown by a recent chocolate-related outbreak of salmonellosis in the UK [138].

## 6. Probiotics: Safety and Quality Control

Safety and efficacy regulation are key factors in the context of “consumer protection law”, and probiotics comprise an excellent example of worldwide attention. There is no news of the raising demand for probiotics due to their well-documented bioactive effects. Delivered through foods, drugs or dietary supplements, infant formula, natural products, among others, different safety requirements exist for each product category [139].

**Figure 3:**
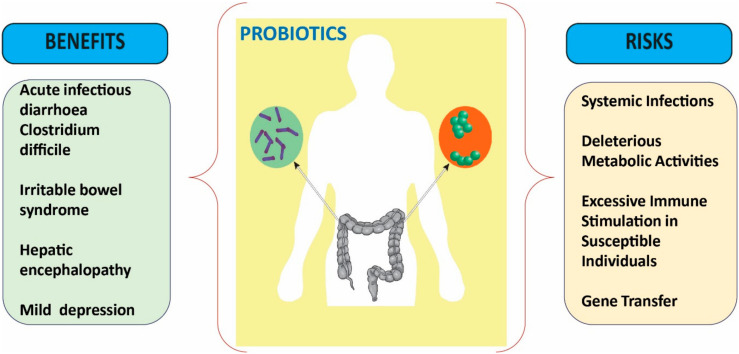
Summarized scheme of the benefits and risks of probiotics.

Currently, it is recognized that a probiotic is accepted as a drug when it cannot be added to food; nonetheless, food probiotics and dietary supplements entail less proof to achieve an authority’s approval in comparison with a long and costly process of drug search/discovery besides constraining the manufacturer’s relevance in clinical studies [140]. Probiotics are of extreme heterogeneity, i.e., two probiotic strains may exactly exert similar clinical effects, but may present distinct safety profiles (Figure 3) [141].

Hence, probiotics shall be evaluated at the strain level. For safety purposes, the FAO/WHO working group recommended that each probiotic strain shall be carefully assessed for toxin production ability, antibiotic resistance, and hemolytic potential, also determining its metabolic activity, and also considering their impact on humans, namely assessing its side effects and post-market surveillance aspects. Not least important is an assessment of their effects in immunocompromised animals, so as to obtain data on its ineffective potential in this type of host [20].

### 6.1. Safety of Probiotics: Side Effects

#### 6.1.1. Systemic Infections

Several case reports describe episodes of infection caused by microorganisms considered as probiotics, e.g., fungemia, bacteremia, sepsis, endocarditis, and others. Fungemia is the most commonly reported single event, with at least 33 reports listing the presence of *S. cerevisiae* or *S. boulardii* in the blood cultures of patients who consumed *S. boulardii*. Bacteremia was reported in 8 cases consuming *Lactobacilli*, including *L. acidophilus*, *L. casei*, and *L. rhamnosus*. Overt sepsis was reported in 9 cases of those who consumed *S. boulardii*, *L. rhamnosus*, *Bacillus subtilis*, *Bifidobacterium breve*, or a probiotic combination [141,142]. Additionally, bacteremia resulting in endocarditis is one of the most important issues in probiotic strain safety. While the *Lactobacillus* infective endocarditis to total bacterial endocarditis ratio varies from 0.05 to 0.4%, there are insufficient case reports in the literature on probiotic bacteremia. *Lactobacillus*-associated bacteremia mostly includes *L. rhamnosus, L. plantarum, L. casei, L. paracasei, L. salivarius,* and *L. acidophilus* [143]. Sanders et al. [144] described 3 risk factors of probiotics identified in these patients: immunocompromised state, weakened intestinal barrier function, and use of a central venous catheter.

#### 6.1.2. Deleterious Metabolic Activities

*Lactobacillus* are mostly associated to D-lactic acidosis, often found in patients with short bowel syndrome. In the case of a colonization with a high load of bacteria, they may lead to diarrhea and small bowel lesions, particularly by bile salt deconjugation and dehydroxylation [145]. In a study with patients with terminal ileostomy, it was stated that consuming fermented dairy products with *L. acidophilus* and *Bifidobacterium*, led to conjugated primary bile salt transformation into toxic free secondary ones through small bowel digestion [146].

In fact, secondary bile salts are dangerous elements, originated by intestinal bacteria on body secretions. These compounds may have carcinogenic potential through interacting with mucus-secreting cells, endorsing proliferation, or even acting as carcinogenesis-triggering agents [147]. There are numerous relevant bacterial enzymes in intestinal carcinogens and toxicant metabolisms (e.g., reductases and hydrolases). Nonetheless and importantly, Kumar et al. [148] showed that probiotics do not possess harmful biochemical activities, such as β-glucuronidase, α-chymotrypsin, β-glucosidase, and N-acetyl-ß-glycosaminidase, frequently related with intestinal disorders (e.g., carcinogens and tumor promoters).

Biogenic amines can be produced and degraded from natural metabolic activity in animals, plants, and microorganisms. Briefly, the elimination of the α-carboxyl group from a precursor amino acid leads to a matching biogenic amine [124]. Occasional cases of food intoxication with histamine, tyrosine putrescine, and cadaverine activity have been credited to *Lactobacillus* [149].

#### 6.1.3. Excessive Immune Stimulation in Susceptible Individuals

Probiotics exert a great influence in the immune system and also a theoretical risk of the potential immune system over-stimulation in susceptible subjects, which can lead to autoimmune response and inflammation. Though, these suppositions have not been long-established in humans [141].

#### 6.1.4. Gene Transfer

It has been acknowledged that probiotic strains may be resistant to some antibiotics and can retain virulence factors and that transferable elements can set these resistances. Hence, there is, theoretically, the possibility of a lateral transfer of genes when using probiotics, which may spread to new and further virulent bacteria. Furthermore, lactic acid bacteria frequently anchor plasmids of diverse dimensions, and, in fact, certain antibiotic resistance determinants placed on plasmids have been found in *L. lactis,* and in several *Lactobacillus* and *Enterococcus* species [150].

So, it is essential to establish both the presence and transfer of bacterial resistance to control and prevent any antibiotic resistance cases [151]. Moreover, plasmid genes are accountable for functions such as bacteriocins production, carbohydrate metabolism, and also for conferring resistance to antibiotics of bacteria (e.g., PAMβ1 encodes resistance to macrolides, lincosamides and streptogramins (MLS)). This was noted in *Enterococcus faecalis* and its capability to transfer genes to other bacteria was confirmed with species of the *Enterococcus, Staphylococcus, Clostridium, Lactobacillus*, and *Bacillus* genera [152]. Likewise, numerous atypical antibiotic resistance-related genes have been listed amongst lactobacilli. For example, in *L. acidophilus*, *L. delbrueckii* subsp. *bulgaricus*, *L. johnsonii, L. reuteri*, and *L. plantarum*, chloramphenicol resistance genes (*cat*; chloramphenicol acetyltransferases) were detected.

Reports also demonstrated the presence of erythromycin resistance genes (responsible for MLS phenotype resistance), yet, the most common resistance genes found in lactobacilli were the tetracycline resistance genes, which typically occur in combination [153]. Infrequently, β-lactam, and aminoglycoside resistance genes have also been detected [154], and there has been no evidence of vancomycin resistance genes in *Lactobacillus* spp.

### 6.2. Quality Control of Probiotics

When looking at the specific characteristics of a probiotic, formerly used as selection criteria, they also aim for quality assurance. The most prominent quality control criteria that must always be controlled and optimized include bile and acid stability; adhesive properties; manufacturing process’ viability and survival; effects on carbohydrates, proteins, and fats; ability to colonize; and immunogenicity. Besides these properties, related to the physiologic properties of the strain, long-term industrial processing and storage conditions also affect probiotic quality. Thus, not only technologic but also functional properties should be considered in quality-control measures [155,156].

Identification methods inappropriately used are a major cause of improper probiotic species designation and probiotic product labelling. These inconsistencies markedly affect probiotics’ efficacy and safety and are likely to exert a negative impact on both health claims and consumer confidence [157].

Different phenotypic and genotypic approaches have been used on probiotic species and strain detection, identification, and characterization [158]. Briefly, nucleic acid-based genotypic approaches can be categorized into (1) hybridization-based techniques, (2) target amplification techniques, and (3) fingerprinting techniques, according to the methodologies used. Molecular techniques have also been used, but all have distinct strengths and weaknesses that consequently affect their applicability [157,158,159,160]. Nonetheless, the use of these techniques is mandatory because phenotypic identifications alone are not reliable enough.

First of all, probiotic strain identification is a critical step, as the correct identification is crucial for diagnostic and epidemiological purposes. Anyway, safety studies (pre-clinical and clinical studies) and those including taxonomy clarification and genome sequencing are also needed to ensure bacterial safety status [161].

## 7. Discussion

The most used probiotics contain strains of *Bifidobacterium* and *Lactobacillus*—the predominant groups of bacteria in the intestinal microbiota. The *S. boulardii* strain also showed beneficial effects. Thus, it is important to take into account the composition of the probiotic administered when interpreting the results on clinical safety and efficacy [162].

The effectiveness of probiotics depends on the species, dose and disease, and the duration of administration depends on the clinical indication. There is evidence that probiotics are effective in acute infectious diarrhea, diarrhea associated with antibiotic treatment, diarrhea associated with *C. difficile* infection, hepatic encephalopathy, ulcerative colitis, irritable bowel syndrome, functional GI disorders, and necrotizing enterocolitis. On the other hand, studies have shown a lack of efficacy in acute pancreatitis and Crohn’s disease [163].

Probiotics help stimulate the synthesis of type A immunoglobulins, and this is the main mechanism by which probiotics are able to support immunity, i.e., to participate in immunomodulation. Studies document the increase in the general production of IgA as well as that of IgA specific to a certain pathogen, as a result of the interaction with probiotics. This action is an extremely important mechanism for anti-infective defense [89].

Studies tend to argue for the superior ability to stimulate IgA immunoglobulin synthesis of probiotic supplement formulas, especially when they are combined with prebiotics. In addition to supporting the synthesis of type A immunoglobulins, probiotics have also been shown to modulate the inflammatory processes and activity of natural killer NK cells, which fight virally infected and cancerous cells [164]. Moreover, probiotics increase the efficiency of the barrier function represented by the intestinal mucosa, therefore reducing the permeability induced by pathogenic microorganisms, but also that associated with inflammation [165].

To ensure health benefits, probiotics can be delivered through foods. A good example of functional food is a probiotic bacteria strains-rich product. The standard of a minimum level of viable probiotic cells ranging from 10^6^ to 10^7^ CFU/mL and the expiration date was also recommended [110]. However, the probiotics used in distinct food matrices is challenging. Distinct species display different sensitivities to substrate acidity, post-acidification in fermented products, dissolved oxygen, metabolism products, temperatures, and GI tract conditions. Bacteria viability and metabolic activity are key aspects on probiotic inclusion in a food raw material, as probiotic bacteria strains need to survive in the food matrix during the shelf life and GI digestion [7].

Dairy-fermented products (i.e., yogurt, probiotic beverages, and cheese-containing LAB) and their constituents (i.e., omega-3 fatty acid, phytosterols, isoflavones, conjugated linoleic acid (CLA), and minerals and vitamins) have a noticeable spot in functional foods formulation [117,166,167]. Moreover, the food industry has been increasingly devoted to the development of new non-dairy foods with probiotics able to surpass the restrictions (e.g., lactose intolerance and cholesterol content) inflicted by milk-based products, that limit their consumption by certain niches of the population [117].

Raw cured products have a very long tradition in Italy, Spain, Germany, France, Netherlands, Austria, and Belgium, with origins in Roman times in the Mediterranean area. Probiotic bacteria strains used in dry fermented meat product production must be able to survive in conditions found in fermented products. Moreover, they should dominate in relation to other microorganisms found in the finished product and need to be well-adapted to fermented product conditions. In a fermented meat environment, the selection of suitable microorganisms is critical because of cell viability, and is more likely strain-dependent [168]. Nonetheless, the probable negative effect on this viability must be valued, especially in the high content in curing salt, the low pH and water activity owed to acidification and drying [124,130]. Furthermore, the content and type of fats, proteins and sugars, and the product pH are features of extreme impact on probiotic growth and survival. Henceforward, product formulation should be manipulated to support a high probiotic efficacy [169]. In this sense, the selected strains to be used in fermented meat product manufacturing need to survive in fermented product conditions to reveal a good ability to adapt to real conditions, to survive fermentation and drying, refrigeration, and storage conditions, rather be able to grow until being able to display health-promoting effects and should have a central place, compared with other microorganisms found in the product (e.g., *Lactobacillus* spp. need close attention in meat fermentation and dry fermented sausage production) [124].

In April 2011, the Agency for Healthcare Research and Quality (AHRQ) of the U.S Department of Health and Human Services (“Safety of Probiotics to Reduce Risk and Prevent or Treat Disease”) published a document evaluating probiotic safety. From 622 studies, in 235 of them merely non-specific safety statements were found, such as “well-tolerated”, while in the remaining ones, the presence/absence of specific adverse events was listed. Built on these reported adverse events, although randomized controlled trials revealed no statistically significantly increase in the relative risk of experienced adverse events, other authors reported that rare adverse events are hard to evaluate [170].

Ideally, probiotic side effects should not exceed the placebo effect (“the zero risk does not exist, and that acceptance of the concept that probiotics may not only have positive effects but potentially also side effects is important”) [171].

According to the FAO/WHO, probiotics may, theoretically, be responsible for 4 types of side effects [20]: systemic infections, deleterious metabolic activities, excessive immune stimulation in susceptible individuals, and, finally, gene transfer [141].

Despite the multiple options available and currently considered, a well-supported report of safe human consumption is the best test for food safety.

## 8. Limitations and Clinical Pitfalls

Evidence on the biological effect of probiotics (colonization of the intestinal mucosa) is limited and controversial. Two recent studies have provided new data showing that although probiotics remain viable throughout the GI tract, they manage to induce only a transient, variable, and individualized response to colonization of the intestinal mucosa [172].

Thus, a personalized, specific approach to the administration of probiotics would be necessary, as empirical supplementation with probiotics may not produce a uniform and persistent effect on the mucosa [173].

A disadvantage of probiotic dietary supplements is a form of preserving bacteria strain, mainly through the lyophilization or microencapsulation. There are many attempts to provide probiotics in the form of tablets, capsules, and lyophilized preparations [174].

Probiotic nutraceuticals have the disadvantage that they can lead to a reduction in functional efficiency due to the exclusion of the potential synergistic effect of food [175,176,177,178]. The alternative method uses starter cultures of probiotic properties in food production as received functional foods exhibit a high nutritional and sensory value. According to Conti-Silva et al. [178], the probiotic LAB contributes the desired odor and flavor of food stuffs through fermentation process, acidifying the products that presents a tangy lactic acid taste and producing aromatic sensory components from amino acids. What is also essential is the protective aspect of the food matrix, e.g., the media of living, active probiotic bacteria cells found within particular nutritional ingredients of food, including fat, sugar, or protein.

Probiotics have been found to delay the recovery of the gut microbiome. Probiotics were ineffective, much faster being the return to the initial state of the microbiome in those who did not take any treatment, as well as in those who received autologous fecal transplantation. Therefore, the context of probiotic use is crucial because, when taken after an antibiotic treatment, they have side effects.

Probiotics are generally considered safe, but caution should be exercised in immunologically vulnerable patients. The long-term effects of probiotic use are unknown and randomized studies are needed.

## 9. Concluding Remarks

Literature data indicate that supplementing a human diet with proper bacteria species showing proven probiotic properties enables a digestive tract to sustain its homeostasis, increasing the tolerance of an organism to unfavorable external stimuli. It facilitates the process of digestion and increases the assimilability of nutritional ingredients as well as shortening the span of recuperation when antibiotic treatment is necessary.

The beneficial effects of probiotics involve several mechanisms, including antagonistic action against specific microorganisms, reducing their number and their effects, direct effect on pathogens metabolism, and stimulating the host’s immunity. Probiotics release antimicrobial and antibacterial agents, improving specific and nonspecific immunity, by activating macrophages, releasing cytokines, and increasing the level of immunoglobulins; ensures adequate intestinal pH by releasing butyric acid, lactic acid, and propionic acid; facilitates the digestion of proteins and lipids and improves lactose intolerance by producing lactase; and has competitive action with pathogens for nutrients and growth factors.

Studies have shown the beneficial roles of probiotics in the prevention and treatment of GI disorders, in the prevention of systemic infections, modulation of the immune system, prevention and treatment of allergies, and in moderating depression and anticarcinogenic effects.

The ideal probiotics should be non-toxic and non-pathogenic bacterial strains that adhere to the intestinal epithelium, thus exerting maximum beneficial effects on the human body. It must also survive in the digestive tract environment, preferably in large numbers, and be able to withstand a low pH and the action of bile salts. Other criteria that the probiotic agent must meet are the ability to produce antimicrobial agents, to modulate the immune system, and to influence the metabolism. Probiotics must be stable and withstand the technological processes of food preparation.

## Figures and Tables

**Table 1:** Summarized data regarding the efficacy/ineffective effects of probiotics in GI diseases.

Gastrointestinal Diseases/Disorders	Effect of Probiotics	Reference
**Efficacy of probiotics in gastrointestinal diseases**		
Acute Bacterial Infectious Diarrhea	↓duration of the disease, ↓the risk of prolongation over four days	[28]
	↓ the number of daily stools	
Traveler’s diarrhea	preventive effect	[29]
Acute viral diarrhea	no significant difference between the group of patients who received the probiotic containing *Lactobacillus rhamnosus* GG and the control group	[30]
Diarrhea associated with antibiotic therapy	prevents or lessens the course of diarrhea	[33,34]
	↓stool frequency, ↑recovery, ↓disease duration	[40]
*Helicobacter pylori* Infection	adjunct to antibiotic therapy to eradicate *Helicobacter pylori* infection	[41]
Ulcerative Colitis, Irritable Bowel Syndrome, Functional Abdominal Pain	it cannot be concluded whether the use of probiotics has a general benefit in the evolution of these diseases.	[43]
	↑remission rate in adults with ulcerative colitis, not maintaining remission, ↓symptoms, ↑ quality of life	[45]
Chronic Constipation	↑ average number of stools per week	[46]
Necrotizing Enterocolitis	↓the risk of severe necrotizing enterocolitis, ↓ mortality	[47]
Hepatic encephalopathy	↓ risk of developing hepatic encephalopathy	[48]
Nonalcoholic Steatohepatitis	↑liver function	[49]
Celiac Diseases, Non-celiac Gluten Sensitivity, Food Allergy	↑protection from gluten-induced pathology	[56]
	↑protection against peanut allergy	[60]
	↑ immune reactions, ↑levels of antibodies	[61]
Symptomatic uncomplicated diverticular disease	the therapeutic effect is not fully known	[66]
**Ineffectiveness of probiotics in gastro-intestinal disease**		
Pancreatitis	Ineffective	[67]
Crohn’s disease	Insufficient evidence	

Symbols: ↑ (increased), ↓ (decreased).
